# The Role of *KCNQ1* Mutations and Maternal Beta Blocker Use During Pregnancy in the Growth of Children With Long QT Syndrome

**DOI:** 10.3389/fendo.2018.00194

**Published:** 2018-04-24

**Authors:** Heta Huttunen, Matti Hero, Mitja Lääperi, Johanna Känsäkoski, Heikki Swan, Joel A. Hirsch, Päivi J. Miettinen, Taneli Raivio

**Affiliations:** ^1^Department of Physiology, Faculty of Medicine, University of Helsinki, Helsinki, Finland; ^2^Children’s Hospital, Pediatric Research Center, University of Helsinki, Helsinki University Hospital, Helsinki, Finland; ^3^Heart and Lung Center, Helsinki University Hospital, Helsinki, Finland; ^4^Department of Biochemistry and Molecular Biology, George S. Wise Faculty of Life Sciences, Institute of Structural Biology, Tel Aviv University, Ramat Aviv, Israel

**Keywords:** growth, ion channels, KCNQ1, long QT syndrome 1, beta blocker

## Abstract

**Objective:**

Two missense mutations in *KCNQ1*, an imprinted gene that encodes the alpha subunit of the voltage-gated potassium channel Kv7.1, cause autosomal dominant growth hormone deficiency and maternally inherited gingival fibromatosis. We evaluated endocrine features, birth size, and subsequent somatic growth of patients with long QT syndrome 1 (LQT1) due to loss-of-function mutations in *KCNQ1*.

**Design:**

Medical records of 104 patients with LQT1 in a single tertiary care center between 1995 and 2015 were retrospectively reviewed.

**Methods:**

Clinical and endocrine data of the LQT1 patients were included in the analyses.

**Results:**

At birth, patients with a maternally inherited mutation (*n* = 52) were shorter than those with paternal inheritance of the mutation (*n* = 29) (birth length, −0.70 ± 1.1 SDS vs. −0.2 ± 1.0 SDS, *P* < 0.05). Further analyses showed, however, that only newborns (*n* = 19) of mothers who had received beta blockers during pregnancy were shorter and lighter at birth than those with paternal inheritance of the mutation (*n* = 29) (−0.89 ± 1.0 SDS vs. −0.20 ± 1.0 SDS, *P* < 0.05; and 3,173 ± 469 vs. 3,515 ± 466 g, *P* < 0.05). Maternal beta blocker treatment during the pregnancy was also associated with lower cord blood TSH levels (*P* = 0.011) and significant catch-up growth during the first year of life (Δ0.08 SDS/month, *P* = 0.004). Later, childhood growth of the patients was unremarkable.

**Conclusion:**

Loss-of-function mutations in *KCNQ1* are not associated with abnormalities in growth, whereas maternal beta blocker use during pregnancy seems to modify prenatal growth of LQT1 patients—a phenomenon followed by catch-up growth after birth.

## Introduction

Defects in ion channels are increasingly implicated in endocrine diseases. For example, dysfunctional or inactive K-ATP channel function due to mutations in *KIR6.2/SUR1* genes leads to congenital hyperinsulinism and/or neonatal diabetes, and defects in potassium channel *KCNJ18* underlie periodic hypokalemic paralysis (1–3). We recently reported that two specific mutations in *KCNQ1*, a gene encoding the alpha subunit of a voltage-gated K+ channel (Kv7.1), result in a gain-of-function in patch clamp analyses and cause pituitary hormone deficiency and maternally inherited gingival fibromatosis (4). This finding was unexpected, since KCNQ1 is traditionally considered as an important regulator of cardiac repolarization rather than one of the key regulators of human growth (5). A prelude for this possibility was achieved already in 2015, however, when Zoledziewska et al. showed that maternally inherited variation in *KCNQ1* was associated with shorter adult height in the Sardinian population, although the mechanism of this finding has not been established (6). In other words, there may be growth-regulating mechanisms unrelated to growth hormone secretion by which KCNQ1 affects height. Indeed, *KCNQ1* is located on chromosome 11p15.5 in a cluster of imprinted growth-regulating genes. The *KCNQ1* locus contains imprinting control region 2, which regulates the imprinting of nearby genes, such as *CDKN1C* (7). Mutations in this imprinted region cause the growth disorders Beckwith–Wiedemann syndrome (BWS) and Silver–Russell syndrome (8). BWS, which is characterized by placental and pre- and postnatal overgrowth, is occasionally caused by maternally inherited mutations which also disrupt *KCNQ1* (9).

According to the parental conflict theory, imprinting has evolved to serve the conflicting interests of the father and the mother; paternally expressed genes promote fetal growth to ensure the viability of the present offspring, whereas maternally expressed genes restrict fetal growth to preserve the mother’s resources to her future offspring (10). Herein, we investigated the growth and endocrine features of patient with long QT syndrome (LQTS) due to loss-of-function mutations in *KCNQ1*. We paid special attention to the relationship between early growth and parent-of-origin of the mutation.

## Subjects and Methods

### Patients

**Figure 1:**
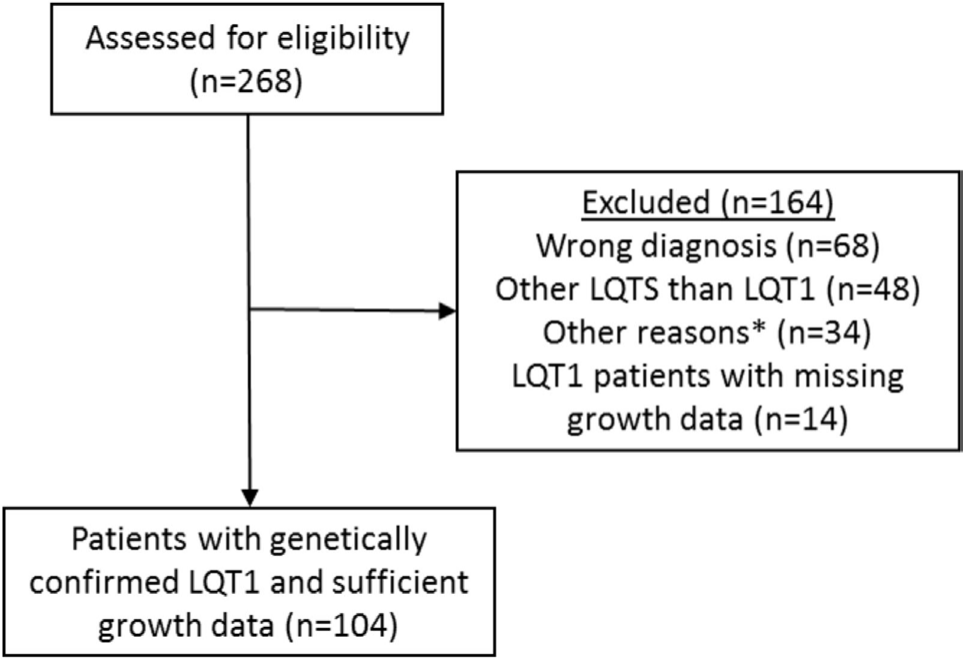
Identification and verification process of the patients with long QT syndrome 1 (LQT1) due to *KCNQ1* mutations at the Children’s Hospital, Helsinki University Hospital. Medical records of 104 patients with genetically verified LQT1 and growth data were identified. See main text for details. *Miscellaneous reasons such as patient’s medical records were not found.

In this retrospective study, the patients diagnosed with LQTS in Helsinki University Central Hospital between 1997 and 2015 were first identified from the electronic health records by using the ICD-10 code I49.8 (other cardiac arrhythmia) which has been used for all LQTS patients. From this population, we excluded those with an erroneous diagnosis (other cardiac arrhythmia than LQTS), other LQTS than LQT1, and those with missing medical or growth data (Figure 1). Overall, 104 patients with genetically diagnosed LQT1 and representative growth data were followed up in the Children’s Hospital from 1997 to 2015, comprising the population of this study. Data collected from all LQT1-patients included gender, specification of the *KCNQ1* gene mutation and its inheritance, treatment modalities, and growth data including measurements from the birth to the adulthood and parental heights. Additionally, all available endocrine data, including the laboratory test results and diagnoses were recorded.

Out of the 104 LQT1 patients, 53 (51%) were males and 7 (6.7%) had been born prematurely. Birth length and weight data were available from 81 patients who were born full term; of them, 29 had inherited the mutation from the father and 52 from the mother. Infancy growth data (at least one length measurement was available from 82 patients, altogether 287 measurements). There were 94 (90%) patients who were recorded to receive beta blocker therapy, 8 symptom-free patients had stopped the medication. The inheritance of *KCNQ1* mutation was documented in all except 2 patients; 67 patients had inherited the mutation from the mother, 33 from the father, and 2 from both parents. The most common mutation was c.1766G > A p.(Gly589Asp) (63%). The molecular genetic findings are described in Table 1.

**Table 1:** Molecular genetic diagnoses of long QT syndrome 1 (LQT1) patients and the structural and rationalized effects of the mutant proteins.

Mutation type	Inherited from (*n*)		Structural effect		Rationalization of effect
	Mother	Father		
c.377A > T p.(His126Leu)	1			Perturbation of *S*1	Interacts with *S*0
c.683 + 5G > A	1				
c.691C > T p.(Arg231Cys)	2			Perturbation of *S*4	Neutralizes one of the positive charges on *S*4
c.805G > A p.(Gly269Ser)		2		Perturbation of *S*5	Steric hindrance with F339 in *S*6
c.830C > T p.(Ser277Leu)	1	1		Perturbation of *S*5	Steric hindrance with A302 in *S*6
c.949G > A p.(Asp317Asn)	2	2		Perturbation of selectivity filter	
c.1022C > T p.(Ala341Val)	2			Perturbation of *S*6	Steric hindrance of S6 kink—helix crossing apposing A341 from neighboring *S*6 (inner gate)
c.1096C > T p.(Arg366Trp)	3	2		Perturbation of pre-helix A: helix A	Possible steric hindrance by W causing some degree of compromised calmodulin (CaM) association and PIP2 binding or gating perturbation (11, 12)
c.1129-2A > G (IVS7-2A > G, FinB)	6	3			
c.1331C > T p.(Thr444Met).		1		Intervening loop	
c.1552C > T p.(Arg518Ter)	2			Truncation of helix B	Predicted abrogation of biosynthesis/assembly (13, 14)
c.1681A > G p.(Arg561Gly)	2			Perturbation of helix C (either coiled coil formation or possibly interactor)	Predicted abrogation of biosynthesis/assembly
c.1766G > A p.(Gly589Asp, FinA)	43	22		Perturbation of helix D	Trafficking affected. characterized in Wiener et al. (15) and Aromolaran et al. (16)
c.1781G > A p.(Arg594Gln)	1			Perturbation of helix D	Discussed in Wiener et al. (15)
*KCNQ1* deletion^a^		1			

*Inheritance pattern was unknown for one patient with FinA and in one with FinB mutation. In addition, two patients had Jervell–Lange–Nielsen syndrome [one patient was homozygous for c.1766G > A p.(Gly589Asp) mutation, and another patient was compound-heterozygous for c.1766G > A p.(Gly589Asp) and p.(Tyr171Ter)]. The KCNQ1 membrane domain has a canonical voltage-gated membrane topology with six transmembrane segments, *S*1–6. Structural studies have shown that many voltage-gated channels have an amphipathic segment called *S*0 that lies in the plane of the inner membrane leaflet, upstream of *S*1. *S*1 through *S*4 comprises the voltage-sensor, and *S5* and *S6* comprise the pore domain. The intracellular C-terminal domain contains a module (helices A–B) to which CaM binds, guiding channel assembly and gating and an additional module with tandem coiled-coils (helices C–D), responsible for tetramerization (15, 17–19)*.

*^a^Details of the deletion not available in medical charts*.

### Statistical Analyses

Values are mean (SD) unless otherwise stated. The analyses were carried out with the SPSS statistical software for Windows, release 22.0 (SPSS, Chicago, IL, USA) and with R^®^ using packages lme4 and lmerTest (20–22). Premature infants were excluded. The one sample *t*-test was used for comparing the height SDS at different ages with the general population. The independent samples *t*-test was employed in analyzing the effect of paternal/maternal inheritance on growth. The linear mixed-model was used for analyzing the associations between birth length, first year growth, beta blocker use during pregnancy and genetic features, and for comparing cord blood TSH values between the subgroups. To account for the sibship between the patients and the differences between the growth of the individuals in the first year model, random intercepts, and slopes were fitted for both the family and the individual. The effects of the beta blocker use during pregnancy combined with the inheritance of the *KCNQ1* mutation were estimated both on birth and per month during the first year. The *p*-values were calculated using Satterthwaite’s degrees of freedom approximation. The statistically significant level was set to *P* < 0.05.

### Informed Consent and Ethical Approval

Since this study is entirely based on health record data, no ethical permission was required according to the Finnish Medical Research Act. The Helsinki University Central Hospital approved the study.

## Results

### Birth Size

Birth length and weight data were available for 81 patients with known maternal or paternal inheritance of the mutation. Those with maternal inheritance of the *KCNQ1* mutation (*n* = 52) were born significantly shorter than those (*n* = 29) with paternal inheritance (−0.70 ± 1.1 SDS vs. −0.20 ± 1.0 SDS, respectively; *P* = 0.046). There was no significant difference in birth weight (3,371 ± 519 vs. 3,515 ± 466 g, respectively; *P* = NS). When the respective comparison was carried out after adjusting for sibship in the linear mixed model, the difference in birth length between the groups remained significant (data not shown). However, some of the patients with maternal inheritance of the *KCNQ1* mutation (and none of those with paternal inheritance) had been exposed to beta blocker treatment prenatally. We, therefore, divided the patients into three groups: those with paternal inheritance of the mutation (PI, *n* = 29), maternal inheritance of the mutation without mother’s beta blocker treatment (MI/BB−, *n* = 33), and those with beta blocker exposure during pregnancy (MI/BB+, *n* = 19). The patients born in the MI/BB+ group were significantly shorter at birth as compared to those with PI, but the birth lengths of MI/BB− and PI groups did not differ (Table 2). The placental weights (*n* = 64) did not differ between the MI/BB+, MI/BB−, and PI groups (512 ± 85 vs. 577 ± 114 g vs. 575 ± 127 g, respectively; *P* = NS).

**Table 2:** Distributions (mean ± SD) of birth length [in standard deviation scores (SDS)] and birth weight in patients with long QT syndrome 1 due to paternally or maternally inherited *KCNQ1* mutations.

	Birth length		Birth weight	
	SDS	*P*	Grams	*P*
Paternal inheritance (*n* = 29)	−0.20 ± 1.0		3,515 ± 466	
Maternal inheritance without beta blocker during pregnancy (*n* = 33)	−0.59 ± 1.1	0.19	3,486 ± 517	0.88
Maternal inheritance and with beta blocker during pregnancy (*n* = 19)	−0.89 ± 1.0	0.017	3,173 ± 469	0.014

*The two maternal inheritance subgroups (without beta blocker and with beta blocker during pregnancy) were compared to the paternal inheritance group with a random effects model*.

### Postnatal Growth

**Figure 2:**
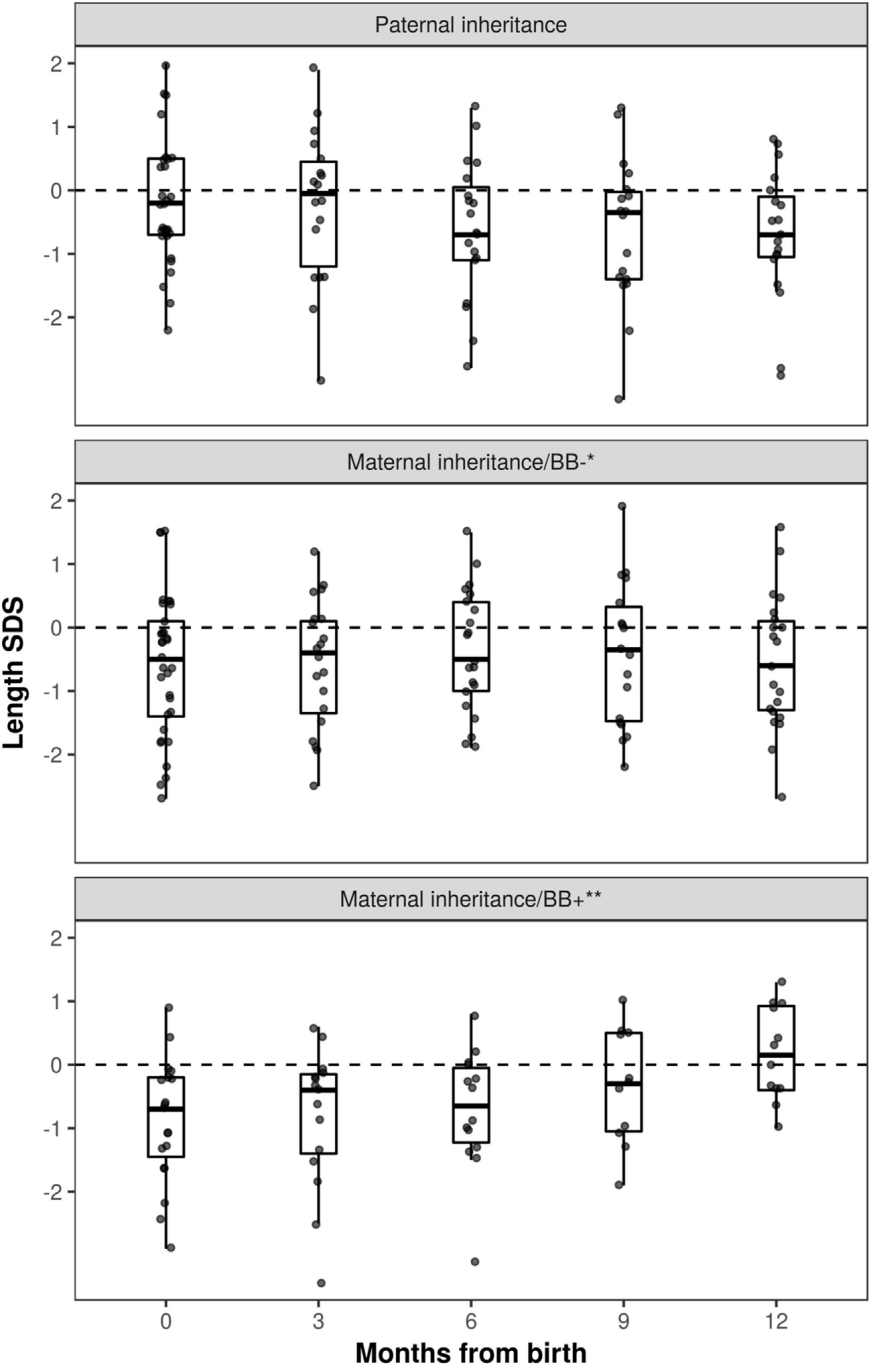
Length standard deviation score (SDS) during the first year of life in long QT syndrome 1 patients. Patients with maternal inheritance of *KCNQ1* mutation and prenatal beta blocker exposure were shorter than those with paternal inheritance at birth (*P* < 0.05), and displayed significant catch-up growth during the first year of life (Δ0.08 SDS/month, *P* = 0.004). *No beta blocker exposure during pregnancy; **prenatal beta blocker exposure.

**Figure 3:**
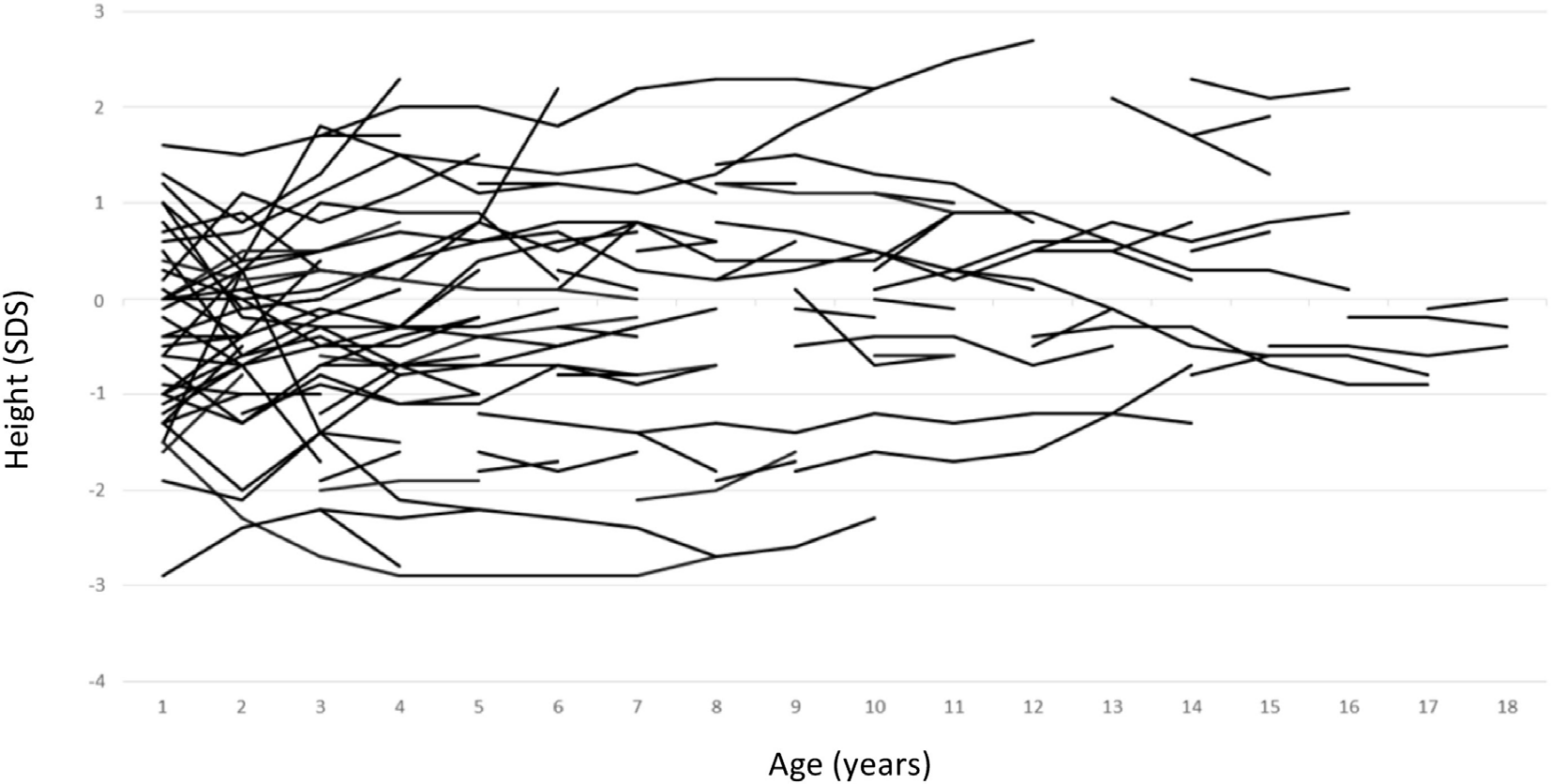
Individual childhood height standard deviation score curves of patients with long QT syndrome 1 due to loss-of-function mutations in *KCNQ1*.

Growth data during the first year of life was available for 82 patients and included 287 measurements. During this period, patients with maternal inheritance and intrauterine beta blocker exposure showed significant catch-up growth (Δ0.08 SDS/month, *P* = 0.004), whereas patients with maternal inheritance and no such exposure grew steadily (Δ0.01 SDS/month, *P* = NS) (Figure 2). Similarly, length SDS in patients with paternal inheritance did not change significantly during the same period (Δ−0.04 SDS/month, *P* = NS). After the first 2 years of life, the patients’ mean height SDS did not significantly differ from the population mean (Figure 3). The two patients with biallelic loss-of-function mutations of the *KCNQ1* had normal birth lengths (−0.7 and 1.5 SDS). Postnatal growth data were available only for one of them and this patient showed normal first year growth (length −0.2 SDS at 1 year of age).

### Other Endocrine Features

As beta blocker treatment appeared to modulate intrauterine growth environment, we compared cord blood TSH levels (*n* = 41) between MI/BB+, MI/BB−, and PI groups. The mean cord blood TSH level in MI/BB− group did not differ from the PI group [6.1 ± 2.3 vs. 8.3 ± 4.7 mU/L, *P* = NS (0.07)]. The lowest mean level was found in the MI/BB+ and the highest mean level in the PI group (4.8 ± 1.6 vs. 8.3 ± 4.7 mU/L, *P* = 0.011). TSH and free T4 levels were measured in 33 (32%) and 24 (23%) LQT1 patients during pediatric follow-up, respectively, and none of the patients were diagnosed with hypo- or hyperthyroidism (data not shown). Insulin-like growth factor I levels were available only from four patients (3.8%) and they were within age- or bone age adjusted reference range. None of the patients received any treatment for endocrine diseases during childhood.

## Discussion

The current work was sparked by our recent finding which showed that two specific mutations in *KCNQ1*, p.(Arg116Leu) and p.(Pro369Leu), underlie growth hormone deficiency and maternally inherited gingival fibromatosis (4). *KCNQ1* is expressed in mouse hypothalamic GHRH neurons, and also in pituitary somatotropes (4), lending credence to the hypothesis that KCNQ1 may act at different levels of the hypothalamic–pituitary axis. KCNQ1 can associate with one of the five KCNE beta subunits that are known to modulate the potassium channel function. KCNE1 is important in the heart, whereas KCNE2 is ubiquitously expressed in different tissues (23). Recently, KCNQ1–KCNE2 complexes were suggested to participate in the regulation of myoinositol transport in the choroid plexus, secretion of insulin, and recently we found that KCNQ1 is implicated in cellular processes, such as spliceosomal snRNP assembly, nuclear import, and intracellular vesicle trafficking (4, 24, 25). The concept of KCNQ1 having a role beyond the regulation of cardiac repolarization is further enforced by the finding that KCNQ1 is implicated in the regulation of beta-catenin signaling in colon cancer (26). We examined endocrine phenotypic features in a large cohort of pediatric patients with loss-of-function mutations in this gene. Our results, however, do not demonstrate clinically relevant associations between the loss-of-function *KCNQ1* mutations and early growth or the endocrine system. These findings suggest that the role of KCNQ1 in the maintenance of the hypothalamic–pituitary axis integrity is not necessarily associated with the electrical activity of the KCNQ1–KCNE channels, and underscore the fact that to date only the codons 116 and 369 link KCNQ1 to pituitary hormone deficiency (4). To our knowledge, mutations in these codons have not been reported in patients with LQT1, and rare amino acid-changing variants are not reported in codons 116 or 369 of *KCNQ1* in Genome Aggregation Database (gnomAD r2.0.2)^1^ or Exome Aggregation Consortium database (ExAC vs. 0.3.1).^2^ Similarly, aberrations in growth or pituitary dysfunction have not been described in patients with the extremely rare short QT syndrome (27, 28), or familial atrial fibrilliation (29) due to activating KCNQ1 mutations, which also corroborates that the electrical activity of KCNQ1–KCNE channels may not be the primary mechanism underlying the KCNQ1-related pituitary dysfynction (4). A clear limitation of our study is, however, paucity of IGF-I levels and other endocrine data.

We found that LQT1 patients exposed to maternal beta blocker medication during pregnancy were smaller at birth than those with paternally inherited mutations, and showed subsequent catch-up growth during the first year of life. Although impaired potassium channel function has been proposed to cause intrauterine growth restriction (30), loss-of-function mutations in *KCNQ1* may not account for this phenotype. Instead, beta blocker use has been previously associated to reduced birth weight in preeclampsia (31–33). Interestingly, ablation of *Kcne2* causes hypothyroidism in pregnant and lactating mice and in their pups (34), yet we did not find any enrichment of thyroid problems in our pediatric LQT1 patient series. Maternal beta blocker use, rather than the parental origin of the *KCNQ1* mutation, was also associated with lower cord TSH levels. To our knowledge, however, beta blocker use during pregnancy has not been reported to modulate offspring thyroid function. On the other hand, cord blood TSH levels are known to be affected by stress-related factors (35), and it is tempting to hypothesize that the effects of such factors on cord blood TSH levels are modified by maternal beta blocker use during the pregnancy. We did not investigate the incidence of disturbances in the glucose homeostasis in our patient cohort, since the frequency of hypoglycemia in Finnish children with LQT1 was very recently reported (36).

In conclusion, loss-of-function mutations in *KCNQ1* have no apparent effects on growth, suggesting that impaired electrical activity of KCNQ1-KCNE channels may not alter growth hormone secretion in a clinically significant manner. Our analyses show, however, that maternal beta blocker use during pregnancy seems to restrict prenatal growth with subsequent and rapid catch-up during the first year of life.

## Ethics Statement

Since this study is entirely based on health record data, no ethical permission was required according to the Finnish Medical Research Act. The Helsinki University Central Hospital approved the study.

## Author Contributions

All authors contributed substantially to manuscript drafting. MH and TR designed the study. HH collected the data. HH, MH, JK, ML, PM, and TR analyzed and interpreted the data and wrote the first version of the manuscript. JH interpreted the predicted effects of KCNQ1 mutations. All authors approved the final version of the manuscript.

## Conflict of Interest Statement

The authors declare that the research was conducted in the absence of any commercial or financial relationships that could be construed as a potential conflict of interest.

## Footnotes

^1^http://gnomad.broadinstitute.org/ (Accessed: April 14, 2018).

^2^http://exac.broadinstitute.org/ (Accessed: April 14, 2018).
